# Genomic insights into the ecological versatility of *Tetracladium* spp.

**DOI:** 10.1186/s12864-025-12146-z

**Published:** 2025-11-05

**Authors:** Anna Lazar, Fabrizio Alberti, George Muscatt, Ryan M. Mushinski, Christopher Quince, Gary D. Bending

**Affiliations:** 1 School of Life Sciences, The University of Warwick, Coventry, CV4 7AL UK; 2 Earlham Institute, Norwich Research Park, Colney Lane, Norwich, NR4 7UZ UK

**Keywords:** Whole-genome, Endophyte, Tetracladium, Comparative genomics, Fungi

## Abstract

**Background:**

*Tetracladium* spp. represent a group of fungi that inhabit various ecological niches, including soil and aquatic environments, where they are considered to have a saprotrophic lifestyle and within plant roots as endophytes. To date, a lack of sequenced *Tetracladium* spp. genomes has inhibited our understanding of their metabolic potential and ecological interactions. In this study, we aimed to elucidate the genetic differences between aquatic saprotrophic and endophytic strains of *Tetracladium* spp. by sequencing and analysing the genomes of *T. maxilliforme* (isolated from *Brassica napus* roots) and *T. marchalianum* (isolated from freshwater), alongside 41 publicly available saprotrophic and endophytic Ascomycetes.

**Results:**

Genomic sequencing revealed that *T. maxilliforme* possesses a genome size of 35.5 Mbp with 9657 predicted genes, while *T. marchalianum* has a genome size of 33.2 Mbp with 15,230 predicted genes. Our analyses primarily focused on carbohydrate-active enzymes (CAZymes). Both genomes possessed the full range of enzymatic machinery for cellulose degradation, as well as the complete repertoire of genes necessary to degrade plant cell walls. Notably, the genomes lacked essential enzymes for lignin degradation or modification. Furthermore, we observed a complete repertoire of known fungal chitin-degrading enzymes in both genomes, which might be related to potential interactions with other fungi. Enzyme composition profiles revealed distinct groupings, with *T. maxilliforme* primarily clustering with endophytic or ecologically versatile species, while *T. marchalianum* was predominantly associated with saprotrophic species. We also identified secondary metabolite biosynthetic gene clusters in both genomes, including several that showed high homology to those of known bioactive compounds.

**Conclusions:**

In summary, our findings offer valuable insights into the genomic adaptations of *Tetracladium* spp. to various ecological niches, highlighting their enzymatic capabilities for carbohydrate degradation and potential interactions within fungal communities.

**Supplementary Information:**

The online version contains supplementary material available at 10.1186/s12864-025-12146-z.

## Background

 Aquatic hyphomycetes, also known as Ingoldian fungi, play a crucial role as decomposers in freshwater ecosystems [1]. Initially discovered in the 1940 s in running freshwater streams, their classification was based on spore morphology, primarily sigmoid or tetraradiate [2]. They produce a diverse set of enzymes capable of breaking down the majority of polysaccharides found in leaves, e.g., pectin, xylan, and cellulose [3]. Upon their introduction to flowing water, these fungi quickly inhabit plant debris, increasing nutrient accessibility for consumption by detritivorous invertebrates [3, 4]. Recognised by their distinctive tetraradiate conidiospores, *Tetracladium* spp. are ubiquitous in aquatic environments [5–9].

Terrestrial observations of aquatic fungi have increased with the availability of DNA sequencing techniques, revealing their presence in soil and plants [10]. Additionally, molecular analysis suggests that some species may have diverse ecological functions [10]. Commonly found in soil, *Tetracladium* spp. have a global distribution, including the Arctic regions [11–17], and have been identified as part of the soil microbiome worldwide, especially in disturbed agricultural and grassland habitats [11, 13, 18, 19]. They are also known as endophytes of plant roots, without host or habitat specificity [14, 20–24]. Our prior research demonstrated that certain species, such as *T. maxilliforme* and *T. furcatum*, exhibit specialisation towards *Brassica napus* root habitats, characterised by a comparatively reduced relative abundance within the soil [25].

The dual ecology of *Tetracladium* spp., specifically whether the same species inhabit both terrestrial and aquatic ecosystems and its role as a plant endophyte remains a subject of debate. Some studies suggest an alternative terrestrial lifestyle serves as a genetic pool for the genus and is linked to the maintenance of high genotypic diversity throughout the year in aquatic ecosystems [5]. The terrestrial endophytic nature of aquatic hyphomycetes was also hypothesised to provide an advantage in decomposing plant litter that reaches freshwater [10]; however, this theory has not yet been proven. Conflicting evidence exists on whether *Tetracladium* spp. endophytes benefit the host, with studies showing both positive and no effects on plant growth [26, 27]. In a study aimed at identifying fungi frequently inhabiting the roots of *Arabis alpina* (Brassicaceae) and examining their potential role in enhancing plant growth and phosphorus (P) uptake, related Helotiales endophytes have been implicated in enhancing P uptake by the plant [28]. Moreover, there is evidence of a co-exclusion relationship between *Tetracladium* spp. endophytes and root pathogenic fungi, and certain *Tetracladium* species display antagonistic effects against bacterial plant pathogens [14, 29].

Despite the ecological importance of *Tetracladium* spp. in freshwater, studies on their terrestrial lifestyle are lacking. While genomic approaches have the potential to offer valuable insights into fundamental biological and evolutionary questions, there is currently limited availability of genomic data for *Tetracladium* spp. Comparative genomics, focusing on diverse metabolic gene clusters, has emerged as a pivotal methodology offering profound insights into the metabolic diversity encoded within genomes. This analytical approach has been widely employed across various ecological niches, encompassing endophytes [30, 31], mycorrhizal fungi [32, 33], pathogens [34, 35], and saprotrophs [36, 37], facilitating a comprehensive understanding of genomic dynamics in these contexts.

In this study, we sequenced and analysed genomes of two *Tetracladium* species. *Tetracladium maxilliforme* originating from *Brassica napus* roots and *Tetracladium marchalianum* isolated from a river. Although both species were initially characterised in freshwater environments [38, 39], they have also been identified in terrestrial habitats [11, 13, 25, 40–42]. Moreover, *Tetracladium maxilliforme* was found to be more abundant in *B. napus* roots than in soil or rhizosphere [14, 25]. Our analysis investigated the repertoire of carbohydrate-active enzymes, lipases, peptidases, and other relevant gene families. We compared these genomic features with those of other ascomycetes to gain insight into their terrestrial ecology. We hypothesise that the diverse enzymatic repertoire of *T. maxilliforme* and *T. marchalianum*, including capabilities for lignocellulose degradation, equips them to thrive in both aquatic and terrestrial environments, supporting their cosmopolitan nature.

## Materials and methods

### Fungal genomes and strains for whole genome sequencing

In the present study, we sequenced the genomes of *T. maxilliforme* and *T. marchalianum*. The former was isolated from surface-sterilised healthy *Brassica napus* roots from a field near Banbury, UK on potato dextrose agar in 2021 (what3words: blip.familiar.mimes, clay-loam soil, pH 6.3, 4.9% soil carbon, collection date: November 2021). The isolate was identified using the ITS2 (internal transcribed spacer) region of the nuclear ribosomal DNA. *T. marchalianum*, originally isolated from freshwater by Ingold from the UK, was purchased from the Westerdijk Fungal Biodiversity Institute (CBS 439.51). Both isolates were grown on potato dextrose agar for six weeks in the dark at 15 °C before further processing.

To compare the newly sequenced *Tetracladium* spp. genomes with those of known fungi, we established a reference fungal genome database sourced from the Joint Genome Institute (JGI) MycoCosm (Suppl. Table 1) [43]. We selected fungal species from the phylum Ascomycota, which have been featured in previous comparative genomic analyses, along with curated ecological information [44–46]. The predicted fungal proteomes associated with these genomes were obtained and downloaded in May 2023. It is important to note that while our selection was drawn from the JGI MycoCosm, it may not encompass all available fungal genomes on the platform.

For comprehensive analysis, fungal genomes were categorised based on their ecological lifestyles, including endophytic, lichenic, mycorrhizal, parasitic, pathogenic, and saprotrophic. To achieve this, we first accessed ecological classifications from the functional groups documented in JGI MycoCosm. For genomes included in previous comparative genomic analyses, we utilised classifications from the respective studies [44, 45, 47]. Genomes lacking classifications were manually curated based on published evidence. In line with prior research, we allowed fungal species to exhibit multiple ecological lifestyles, provided that there was supporting evidence. Ultimately, our selection of genomes encompassed those categorised as saprotrophic, endophytic, or displaying a combination of ecologies that incorporate these lifestyles.

### Genome sequencing, assembly, and annotation

To sequence the *Tetracladium* spp. genomes, we first collected 0.2 g of fungal mycelia and then extracted genomic DNA (gDNA) using the DNeasy PowerSoil Pro Kit (Qiagen, Germany) according to the manufacturer’s instructions. The quantity and quality of gDNA were assessed using the Qubit dsDNA HS Assay Kit (Thermo Fisher Scientific, MA, USA). We then prepared the sequencing library using the Nanopore Ligation Sequencing Kit V14 (Oxford Nanopore Technologies (ONT), UK) according to standard instructions. We sequenced the libraries on MinION flowcells (R10.4.1, ONT, UK). Basecalling was performed using Guppy (v7.1.4) after sequencing with the 400 bps–5 kHz and super-accurate base calling settings with no base modifications in the MinKNOW software (v23.07.15, ONT, UK). The quality of the reads was assessed using *pycoQC* (v2.5.0.17) [48]. Genomes were *de novo* assembled using *Flye* (v2.9.2) without scaffolding to avoid gaps. The quality of the assembly and the completeness of the genomes were tested using the BUSCO Fungi odb10 single-copy orthologs database (v5.4.7) and screened with *tiara* (v1.0.3) [49].

For better gene prediction, we extracted and sequenced total RNA from the fungal isolates. We used 0.2 g of mycelium with the Monarch^®^ Total RNA Miniprep Kit (New England Biolabs, UK) according to the standard instructions. RNA quality was assessed using the Qubit RNA BR Assay Kit (Thermo Fisher Scientific, MA, USA). Total mRNA was sequenced by Novogene on an Illumina NovaSeq platform using paired-end sequencing with a read length of 150 bp. The library was sequenced following end-repair, A-tailing, adapter ligation, size selection, amplification, and purification. Following sequencing, reads containing adapters, reads containing more than 10% uncertain bases, and reads containing low-quality bases (Qscore ≤ 5) were filtered. Putative proteins were predicted and annotated using the *funannotate* prediction and annotation pipeline (v1.8.16) [50] following the standard instructions based on the protein databases of Pfam, gene2product, InterPro, dbCAN, MEROPS, MIBiG, UniProt, Gene Ontology, RepeatsDB, Phobius, and EggNOG.

In our comparative analyses of all genomes, we focused on Carbohydrate-Active Enzymes (CAZymes), lipases, proteases, transporters, and small secreted proteins. To identify CAZymes, fungal proteomes were queried against an HMM (Hidden Markov Model) profile database constructed from CAZy sequences sourced from the dbCAN2 database (downloaded from: https://bcb.unl.edu/dbCAN2/download/Databases/V11/dbCAN-HMMdb-V11.txt) [51] using HMMer (v3.3.2) [52]. Protein hits were filtered using an e-value threshold of 1e^−15^ and an alignment threshold of 50% of the domain query.

Fungal proteomes were queried against the MEROPS peptide database (accessed in July 2023 from: https://ftp.ebi.ac.uk/pub/databases/merops/current_release/merops_scan.lib) using BLASTp, and protein hits were filtered using an e-value threshold of 1e^−10^. Lipases were identified by querying fungal proteomes against the Lipase Engineering Database (v4.1.0), with DIAMOND (v2.1.8) [53], employing the option " -sensitive,” and filtering hits with an e-value threshold of 1e^−5^.

Transporters were identified by querying fungal proteomes against the TCDB transporter database (accessed in November 2023) [54] using BLASTp, with protein hits filtered using an e-value threshold of 1e^−10^. Secreted proteins were identified through a custom pipeline as previously reported [55]. This involved signal peptide identification with SignalP (v4.1) [56], extracellular localization prediction with WoLFPSort (v0.2) [57], transmembrane helix identification with TMHMM (v2.0) [58], secretory pathway association inference with TargetP (v2.0) [59], and confirmation of the absence of a KDEL motif in the C-terminal region with PS-SCAN (v1.86) [60].

Small-secreted proteins were identified as proteins < 300 amino acids with no CAZyme, lipase, protease, or transporter annotation. Functional annotation of putative small-secreted proteins was achieved using PfamScan (v1.6) [61] with an e-value threshold of 1e^−15^ and an alignment threshold of 50% of the domain query. For each identified functional protein category, a single best hit per locus was retained by selecting the hit with the smallest e-value and greatest alignment of the domain query.

Fungal proteomes with predicted functions were compared in R (v4.2.2) [62]. Heatmaps and hierarchical clustering analysis were generated using *pheatmap* (v1.0.12) [63]. Uniform Manifold Approximation and Projection for Dimension Reduction (UMAP) was used to assess the similarity of transporters and small secreted proteins in the genomes using *umap* (v0.2.10.0). The bar plot, assessing overall gene counts, and the ordination plots were generated with ggplot2 (v3.4.4) [64].

Secondary metabolite genome mining was performed using antiSMASH (v7.1.0, fungal version) [65] with KnownClusterBlast, ClusterBlast and MIBiG cluster comparison features enabled using the “relaxed” detection strictness.

## Results

### Genomic features of the newly sequenced Tetracladium maxilliforme and Tetracladium marchalianum genomes

In this study, we sequenced the genomes of two isolates *T. maxilliforme* and *T. marchalianum*. The newly sequenced genomes displayed similar sizes, measuring 35.5 Mbp and 33.3 Mbp, respectively. The N50 value was determined to be 1.4 Mbp for *T. maxilliforme* and 0.034 Mbp for *T. marchalianum*. Additionally, the genome coverage achieved was 23x for *T. maxilliforme* and 15x for *T. marchalianum* (Table 1). The initial drafts of the *T. marchalianum* and *T. maxilliforme* genomes had 133 and 8 bacterial sequences, respectively, identified by Tiara. These were removed, the genomes were reassembled, and further checks confirmed that there was no bacterial contamination. To assess the quality and completeness of the genomes, we screened against the BUSCO database and found 99.1 (C:99.1% [S:98.3%, D:0.8%], F:0.0%, M:0.9%) and 91.6 completeness (C:91.6% [S:90.5%, D:1.1%], F:3.0%, M:5.4%) for *T. maxilliforme* and *T. marchalianum*, respectively (Table 1). Furthermore, our analysis provided insights into the gene content of the *Tetracladium* spp. isolates. Using the *funannotate* pipeline we identified and functionally annotated 9651 and 15,230 predicted genes in the *T. maxilliforme* and *T. marchalianum* genomes, respectively. Of these genes, 4973 and 7030 were annotated using *funannotate*, respectively. The BUSCO scores for the predicted transcriptome were C:88.4% [S:87.9%, D:0.5%], F:1.2%, M:10.4% for T. *maxilliforme*, and C:89.6% [S:88.8%, D:0.8%], F:4.0%, M:6.4% *T. marchalianum*.

**Table 1:** Genomic features of *Tetracladium maxilliforme* and *Tetracladium marchalianum*

	*Tetracladium maxilliforme*	*Tetracladium marchalianum*
Assembled genome size (Mbp)	35.5	33.3
Sequencing read coverage depth	23	15
Number of contigs	162	1706
Contig N50 (Mbp)	1.4	0.034
Contig L50	9	284
GC content	54.5	47
BUSCO completeness %	99.1	91.6
BUSCO coplete single-copy %	98.3	90.5
BUSCO coplete duplicated %	0.8	1.1
BUSCO complete + partial %	99.1	94.6
Total number of predicted genes	9657	15230
Number of predicted mRNA	9531	15032
Mean gene length (bp)	1558	1292
Total number of annotated proteins	4419	5925

**Fig. 1:**
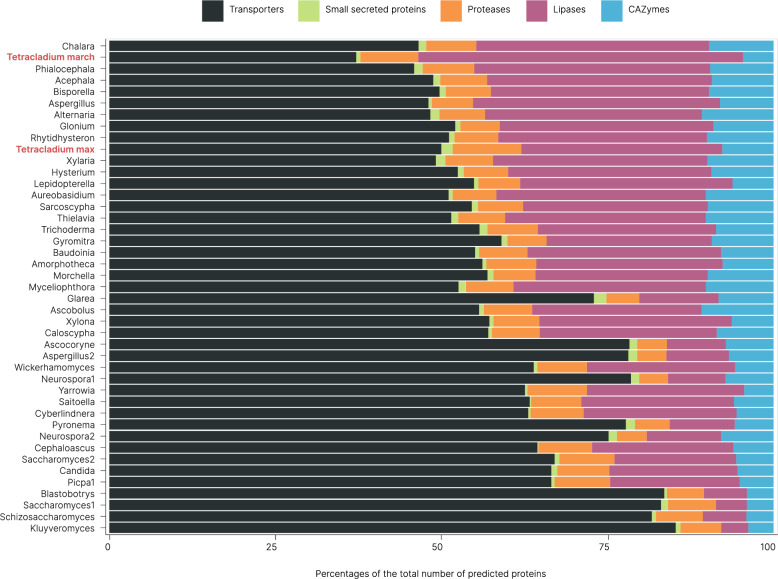
Proportion of predicted proteins classified as CAZymes, lipases, peptidases, small secreted proteins, and transporters relative to the total annotated proteins in 43 ascomycotan genomes

Of the 43 genomes used for comparative genomic analysis, we assigned 65% of the genomes as saprotrophic, 7% as endophytes, and 28%, including the two *Tetracladium* spp. genomes, as multiple ecologies (Suppl. Table 1). The 43 genomes exhibited an average of 299 CAZymes, 1017 lipases, 255 peptidases, 34 small secreted proteins, and 2073 transporters. Notably, *T. marchalianum* had proportionally the least transporters but the most lipases (Fig. 1). Specifically, we annotated 384 and 324 genes from the CAZyme database; 1504 and 3455 genes from the Lipase Engineering Database; 487 and 572 genes from the MEROPS database; 84 and 42 small secreted proteins; and 2483 and 2622 genes from the transporter database for *T. maxilliforme* and *T. marchalianum*, respectively.

### Identification and analyses of CAZymes in the genomes

*T. maxilliforme* clustered with *Aureobasidium pullulans*, *Aspergillus niger*, *Glonium stellatum*, and *Hysterium pulicare* based on their CAZyme classes. Conversely, *T. marchalianum* appeared to be more closely associated with *Trichoderma reesei* based on the clustering observed in the heatmap (Suppl. Figure 1). These distinct groupings exhibited variations in their glycoside hydrolase (GH) protein counts. Additionally, differences were observed in the counts of carbohydrate esterase (CE) and auxiliary activity (AA) proteins among the identified groups. Both groups had predicted proteins in all CAZyme classes, except for carbohydrate-binding modules (CBM) (Suppl. Figure 1).

**Fig. 2:**
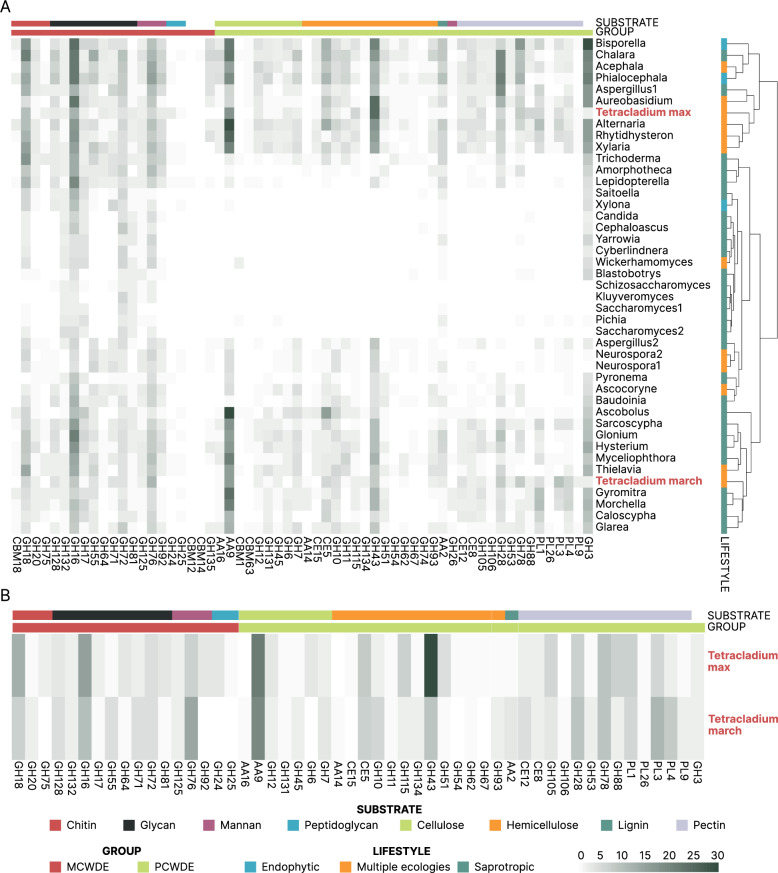
Cell-wall degrading enzyme profiles of the annotated genomes based on carbohydrate-active enzyme families. **A** – Hierarchical clustering analysis on the *Tetracladium maxilliforme* (Tetracladium max), *Tetracladium marchalianum* (Tetracladium march) and the Ascomycotan genomes. The clustering was based on the abundance and composition of carbohydrate-active enzyme (CAZyme) families associated with cell wall degradation. Shading darkness shows protein copy numbers. Coloured boxes next to the short genome names show lifestyle. The top colour bar denotes enzyme substrates. The bottom colour bar denotes microbe (red) or plant (green) cell wall degradation. **B** – Protein copy counts of CAZYyme families associated with cell wall degradation for the *Tetracladium* spp. genomes. Shading darkness corresponds to copy numbers. The top colour bar denotes enzyme substrates. The bottom colour bar denotes fungal (red) or plant (green) cell wall degradation

To gain deeper insights into the carbohydrate degradation capabilities of *Tetracladium* spp., we focused on the enzymes involved in the degradation of microbial and plant cell walls. Initially, we identified CAZyme families associated with microbial and plant cell wall degradation (MCWD and PCWD) as highlighted by Yang et al. [30]. These CAZymes play a crucial role in various processes, such as endophytic fungi penetrating their hosts, biocontrol fungi inhibiting pathogenic fungi, and the decomposition of dead plant material. We identified 194 and 170 predicted cell wall-degrading proteins in the genomes of *T. maxilliforme* and *T. marchalianum*, respectively. Hierarchical clustering of these profiles showed *that T. maxilliforme* clustered with *Alternaria alternata*, *Rhytidhysteron rufulum*, and *Xylaria hypoxylon*. *T. marchalianum* clustered with *Gyromitra esculenta*, *Morchella importuna*, *Caloscypha fulgens*, and *Glarea lozoyensis* (Fig. 2A).

Notably, both *Tetracladium* spp. genomes exhibited a comparable proportion of cell wall degradation enzymes, with 16% of annotated wall-degrading CAZymes dedicated to microbial cell wall degradation. We identified most of the primary CAZyme classes responsible for degrading fungal cell wall components. For chitin degradation, GH18 was the most abundant enzyme family in both *T. maxilliforme* with 11 copies and *T. marchalianum* with 6 copies. Similarly, in the context of glycan degradation, GH16 exhibited prominence across both genomes, with 13 copies in the *T. maxilliforme* and 9 copies in the *T. marchalianum* genome. Moreover, the examination of mannan-degrading enzyme families revealed that GH76 was the most abundant, with *T. maxilliforme* possessing 6 copies and *T. marchalianum* featuring 12 copies. In contrast, considering the degradation of bacterial peptidoglycan, only *T. maxilliforme* demonstrated potential enzymatic activity, as indicated by the presence of 3 copies of GH24 and 1 copy of GH25 within its genome (Fig. 2B).

Furthermore, a significant portion of the identified cell wall-degrading enzymes were associated with plant cell wall degradation, constituting 84% of the cell wall-degrading domains. The most abundant CAZyme classes linked to plant cell wall degradation included AA9 for cellulose (19 copies in the *T. maxilliforme* and 17 copies in the *T. marchalianum* genome), and CE5 (*T. maxilliforme*: 7 copies, *T. marchalianum*: 6 copies), GH115 (*T. maxilliforme*: 6 copies, *T. marchalianum*: 2 copies) and GH43 (*T. maxilliforme*: 27 copies, *T. marchalianum*: 8 copies) for hemicellulose (Fig. 2B).

Concerning the degradation of pectin, both genomes exhibited a diverse array of enzyme families. Notably, GH28, with 6 copies in *T. maxilliforme* and 8 copies in *T. marchalianum*, alongside GH78, displaying 8 copies in both genomes, emerged as prominent contributors to this process. Additionally, enzyme families PL1 and PL3 were found to be significant, with *T. maxilliforme* featuring 6 copies of PL1 and 5 copies of PL3, while *T. marchalianum* had 5 copies of PL1 and 10 copies of PL3. Furthermore, PL4, although less abundant, were present, with *T. maxilliforme* possessing 2 copies and *T. marchalianum* featuring 7 copies (Fig. 2B). Notably, *T. maxilliforme* displayed a higher copy number of hemicellulose-degrading CAZyme families (a total of 53 in the *T. maxilliforme* and 29 in the *T. marchalianum* genome), whereas *T. marchalianum* exhibited more pectin-degrading domains (a total of 47 in the *T. maxilliforme* and 57 in the *T. marchalianum* genome) (Fig. 2B).

We analysed specific lignocellulolytic enzymes utilising enzyme classification numbers derived from the functional annotations of *Tetracladium* spp. We found 60 annotated genes in *T. maxilliforme* and 64 in *T. marchalianum* that were associated with lignocellulolytic enzymatic activity (Table 2). There was a predominant representation of cellulose-degrading enzymes in both genomes, accounting for 68% of lignocellulolytic gene counts in *T. maxilliforme* and 63% in *T. marchalianum*. Furthermore, we identified an abundance of beta-glucosidase (EC 3.2.1.21) in both genomes. Additionally, endoglucanases (EC 3.2.1.4) and exoglycanases (EC 3.2.1.91) were observed, indicating a comprehensive repertoire of enzymatic machinery involved in cellulose degradation in both *T. maxilliforme* and *T. marchalianum* (Table 2). The hydrolysis of hemicellulose requires multiple enzymes operating at various levels within the hemicellulose matrix. This cooperative activity is essential due to its interconnectedness with other components of the plant cell wall. Predominantly, two classes of enzymes play pivotal roles in hemicellulose degradation: endo-1,4‐β‐xylanase (EC 3.2.1.8) and exo‐1,4‐β‐xylosidase (EC 3.2.1.37) [66]. Notably, in the genomic inventory of *T. maxilliforme*, 3 copies of both the endoxylanase gene and the beta-xylosidase gene were identified. Conversely, *T. marchalianum* exhibited six copies of the endoxylanase gene, while the beta-xylosidase gene was absent from its genomic repertoire (Table 2). We found that neither of the *Tetracladium* spp. genomes had the essential enzymes for lignin degradation or modification. However, both *T. maxilliforme* and *T. marchalianum* had copies of glyoxal oxidase (EC. 1.2.3.15), an auxiliary enzyme in lignin modification [67] (Table 2).

**Table 2:** Protein copy counts of lignocellulolytic enzymes in the *Tetracladium maxilliforme* and *Tetracladium marchalianum* genomes

EC number	Enzyme name	Count Tetracladium maxilliforme	Count Tetracladium marchalianum	Degradation pathway
3.2.1.21	Beta-glucosidase	27	23	Cellulose
3.2.1.4	Endoglucanase	10	12	Cellulose
3.2.1.91	Exoglucanase	4	5	Cellulose
3.1.1.72	Acetyl-xylanesterase	2	2	Hemicellulose
3.1.1.73	Ferulic acid esterase	3	5	Hemicellulose
3.2.1.139	Alpha-glucuronidase	1	0	Hemicellulose
3.2.1.37	Beta-xylosidases	3	0	Hemicellulose
3.2.1.55	Alpha-arabinofuranosidase	5	0	Hemicellulose
3.2.1.8	Endoxylanase	3	0	Hemicellulose
1.2.3.15	Glyoxal oxidase	2	0	Lignin
1.10.3.2	Laccases	0	0	Lignin
1.11.1.14	Lignin peroxidase	0	0	Lignin
1.11.1.13	Manganese peroxidase	0	0	Lignin
1.11.1.16	Versetile peroxidase	0	0	Lignin
1.11.1.19	Dye decolorizing peroxidase	0	0	Lignin

**Fig. 3:**
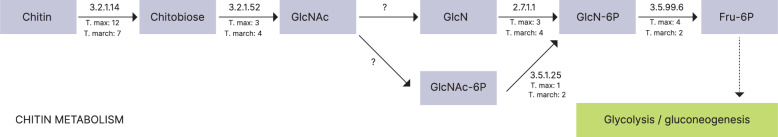
Chitin degradation pathway based on the KEGG (Kyoto Encyclopedia of Genes and Genomes) amino sugar and nucleotide sugar metabolism (map00520) pathway, with protein counts for *Tetracladium maxilliforme* and *Tetracladium marchalianum*. The numbers above the arrows are Enzyme Commission (EC) numbers with copy numbers for each genome under the arrows

To better understand the potential degradation of chitin, we focused on all known enzymes involved in the amino sugar and nucleotide sugar metabolism pathway (KEGG glz00520). This pathway, documented in the Kyoto Encyclopedia of Genes and Genomes (KEGG) [68], is conserved across all organisms, including fungi. We found that both genomes of *Tetracladium* spp. exhibited an abundance of all essential enzymes for chitin degradation, with one notable exception: the absence of a deacetylase (EC 3.5.1.33 N-acetylglucosamine deacetylase) and/or kinase (EC 2.7.1.59 N-acetylglucosamine kinase), which are exclusive to bacteria (Fig. 3).

### Comparative genomic analyses of peptidases, lipases, transporters, and small secreted proteins and assessing the loss of metabolic pathways associated with host dependency

The genomes of *Tetracladium* spp. exhibited many serine peptidases, forming a distinctive cluster based on the abundance and composition of peptidases. This cluster shared similarities with *Chalara longipes*, particularly the heightened presence of aspartic peptidases (Suppl. Figure 2). Additionally, the larger cluster to which the *Tetracladium* spp. genomes belonged to encompass the majority of endophytic and multiple ecology genomes (Suppl. Figure 2).

We conducted a comparative analysis of lipase compositional profiles within the genomes of *Tetracladium* spp. in relation to other fungi. We found that *T. maxilliforme* clustered with *A. pullulans*, *H. pulicare*, and *X. hypoxylon*, whereas *T. marchalianum* formed a distinct group alongside *C. longipes*, *Phialocephala scopiformis*, *Aspergillus niger*, *Acephala macrosclerotiorum*, and *Bisporella* sp. These groups exhibited differentiation based on the abundance of N-terminal cap lipase, with *T. maxilliforme* displaying lower levels compared to *T. marchalianum* (Suppl. Figure 3). The larger cluster to which the *Tetracladium* spp. genomes belonged, similarly to the peptidases, included most of the endophytic and multiple ecology genomes (Suppl. Figure 3).

The UMAP plots revealed noteworthy similarity in the transporters and small secreted proteins among the genomes of *Tetracladium* spp. (Suppl. Figure 4A and 4B). The *Tetracladium* spp. genomes formed a distinct group with thirteen of the genomes based on their transporters (Suppl. Figure 4A). Most of these genomes were characterized as endophytic or had multiple ecological lifestyles such as *G. lozoyensis*, *H. pulicare*, *X. hypoxylon*, *G. stellatum*, *A. alternata*, and *R. rufulum*. In contrast, the annotated genomes exhibited general similarity in their small secreted protein profiles (Suppl. Figure 4B).

Identification of missing genes associated with vital biological processes offers valuable insights into the essential nutrient requirements of fungi. The genome sequence of the model arbuscular mycorrhizal *Rhizophagus irregularis* marked a significant milestone in understanding host dependency in fungi. This analysis revealed the absence of key metabolic enzymes, such as thiamine synthase and type I fatty acid synthase (FAS [69, 70]. Recent findings indicating lipid transport from plants to arbuscular mycorrhizal fungi align with the fungi’s inability to produce fatty acids independently [71]. To identify FAS genes and thiamine biosynthetic pathway genes, we searched the annotated genomes for these sequences identified in *Saccharomyces cerevisiae* as described by Kobayashi et al., 2018 [72]. Of the nine FAS genes, five were not found in the *T. maxilliforme* genome, with four genes missing from the *T. marchalianum genome* (Suppl. Table 2). The *T. maxilliforme* genome was missing the FAS2 (Type 1), HFA1, MCT1, OAR1, HTD2 (Type 2) genes. The same genes were absent from the *T. marchalianum* genome except for MCT1. The absence of some genes associated with thiamine biosynthesis was observed in both the *T. maxilliforme* and *T. marchalianum* genomes (Suppl. Table 2).

Given the limitations of automated annotation pipelines, we conducted a manual BLAST search to verify the absence of these genes. This additional analysis revealed that some of the initially undetected genes were, in fact, present in the genomes but may have been overlooked due to fragmentation, misannotation, or divergence from reference sequences. The BLAST results confirmed the presence of homologous sequences for FAS2 for both genomes (Suppl. Table 2).

### Identification of secondary metabolite biosynthetic gene clusters in the genomes

We found that the genomes of *T. maxilliforme* and *T. marchalianum* contained 51 and 22 secondary metabolite biosynthetic gene clusters (BGCs), respectively (Table 3). Among these, non-ribosomal peptide synthetases (NRPSs) appeared to be the most prevalent class of BGCs in *T. maxilliforme*, followed by NRPS-like and polyketide synthase (PKS) BGCs. Conversely, NRPS-like, PKS and ribosomally synthesised and post-translationally modified peptides (RiPPs)-like were the most abundant classes of BGCs in *T. marchalianum*. Notably, we found BGCs that showed high homology to those of known bioactive natural products (Suppl. Table 3), such as for the cyclic depsipeptides aureobasidin A1 (antifungal antibiotic) and enniatins (mycotoxin), for the PK/NRP hybrids ilicicolin H (antifungal antibiotic) and for the terpene-polyketide hybrids ascochlorin (antiviral, anti-inflammatory, cytotoxic) in *T. maxilliforme*; and for the NRP xenortide A (antimicrobial) in *T. marchalianum*. Additional BGCs from the two genomes showed similarity to other known BGCs, such as for the terpenes clavaric acid (antitumor) and squalestatin S1 (cholesterol lowering agent), the PK/NRP hybrid fusaristatin A (antifungal), the polyketide mellein (antibacterial, antifungal, antiviral, herbicidal), and the NRPs metachelin (siderophores), phenguignardic acid (herbicidal), and choline (essential nutrient) in *T. maxilliforme*; and for the PK solanapyrone A (herbicidal), alkaloids chaetoglobosins (antifungal), and NRPs metachelin, phenguignardic acid and choline in *T. marchalianum*.

**Table 3:** Secondary metabolite biosynthetic gene clusters (BGCs) predicted to be present within the genomes of *Tetracladium maxilliforme* and *Tetracladium marchalianum* based on antiSMASH. Key for BGC classes: NRPS= non-ribosomal peptide synthetase; PKS= polyketide synthase; RiPP= ribosomally synthesised and post-translationally modified peptide

BGC class	*Tetracladium maxilliforme *	*Tetracladium marchalianum*
NRPS	14	3
NRPS-like	12	7
PKS	9	5
Hybrid PKS/NRPS	5	-
Terpene	6	1
RiPP	1	-
RiPP-like	1	5
Betalactone	1	-
Isocyanide-NRP	1	1
Indole	1	-
Total BGCs	51	22

## Discussion

This study presents the genomic features of *Tetracladium maxilliforme* and *Tetracladium marchalianum*, revealing similar genome sizes of 35.5 Mbp and 36.6 Mbp, respectively. The difference in predicted gene numbers between *T. maxilliforme* (9,651 genes) and *T. marchalianum* (15,230 genes) is likely attributable to the fragmentation of the *T. marchalianum* genome assembly. Both species displayed an enriched repertoire of CAZymes, lipases, peptidases, small secreted proteins, and transporters than the average of the 43 Ascomycete genomes they were compared to. Further exploration of carbohydrate degradation capabilities highlighted distinct clustering patterns and variations in glycoside hydrolase protein counts among *Tetracladium* spp. The genomes harboured numerous enzymes involved in fungal and plant cell wall degradation. They contained a repertoire of cellulose-degrading enzymes. Chitin degradation capabilities were identified. Comparative analyses revealed clustering patterns in the lipase profiles, with *T. maxilliforme* and *T. marchalianum* forming distinct clusters. Biosynthetic gene clusters (BGCs) for secondary metabolites were also identified, including those with homology to known BGCs that produce bioactive natural products with antifungal, antibiotic, antiviral, and cytotoxic activities. The genomic analysis of *Tetracladium maxilliforme* and *Tetracladium marchalianum* in this study provides insights into their genetic characteristics and functional potential. In our analysis, we observed a decreasing trend in BUSCO completeness scores as we moved from broader fungal datasets to more taxon-specific sets, highlighting the limitations of existing reference databases in accurately representing *Tetracladium* due to its uncertain phylogenetic placement and the lack of closely related species in OrthoDB (Supplementary Table 4). The predicted gene BUSCO completeness scores were approximately 10% lower than the assembly completeness for both genomes, indicating potential gene losses or misannotations. To enhance the accuracy and reliability of gene annotations, it is essential to complement automated predictions with manual curation and cross-validation using experimental data where possible. This integrative approach mitigates the risk of omitting biologically significant genes, ensuring a more comprehensive understanding of the genomic landscape.

### Proficient plant cell wall degradation, excluding lignin

The clustering patterns identified within the CAZyme classes with those of other fungal species offer insights into potential ecological niches and functional similarities. *T. marchalianum* was found to cluster with the saprotrophic *Trichoderma reesei* which serves as a crucial model organism for studying the enzyme systems responsible for lignocellulose degradation [73] and is known for its proficiency in crystalline cellulose degradation [74]. Meanwhile, *T. maxilliforme* was grouped with *Aureobasidium pullulans*, *Aspergillus niger*, *Glonium stellatum*, and *Hysterium pulicare* based on their overall CAZy abundance and composition profiles. These species are recognized for their robust capabilities in degrading plant cell walls. *A. pullulans*, a generalist yeast, is commonly found in various dead plant materials and can inhabit plants, sometimes leading to diseases [75]. Additionally, *G. stellatum* and *H. pulicare* both demonstrate an affinity for wood or bark habitats [76, 77].

Considering their set of cell wall degrading enzymes, *T. maxilliforme* shared similarities with genomes linked to diverse ecological niches, whereas *T. marchalianum* clustered with genomes associated predominantly with saprotrophic lifestyles, consistent with their respective original isolation environments. Although both species have been previously characterised as endophytes based on ITS amplicon sequencing [13, 25, 40], it is crucial to highlight that the isolates used in this investigation originate from distinct ecological contexts. Specifically, the *T. maxilliforme* strain was isolated from root samples, whereas *T. marchalianum* was isolated from a freshwater habitat.

A more in-depth examination of carbohydrate degradation capabilities, particularly in cell wall degradation enzymes, highlighted the potential importance of *Tetracladium* spp. in ecological processes such as endophytic interactions and plant material decomposition. The genus demonstrated higher hemicellulose degradation enzyme copy numbers, particularly those of glycoside hydrolases class 43, and showed potential for pectin degradation. Within CWDEs, the significance of pectin-degrading enzymes stands out as vital for fungal infection in plants. These enzymes play a crucial role by facilitating the action of cellulases and hemicellulases on exposed cell wall components, paving the way for their subsequent degradation [78]. This mechanism facilitates the degradation of plant cell walls, and can potentially indicate *Tetracladium* spp.’s ability to function as an endophyte, establishing symbiotic relationships within plant tissues. 

*Tetracladium* spp. had particularly high AA9 (lytic cellulose monooxygenase, formerly GH61) counts within the cellulose-degrading enzyme groups. The group generates new initiation sites for conventional cellulases and directly oxidases cellulose, leading to the mechanical weakening of the ultrastructure [79]. Analysis of the main lignocellulolytic enzymes highlights the significant role of *Tetracladium* spp. in cellulose degradation, with a predominant representation of cellulose-degrading enzymes. In fungi, the cellulase system comprises three hydrolytic enzymes: β-glucanase, responsible for cleaving β-linkages randomly in amorphous cellulose regions; exoglucanase, which liberates cellobiose from either the nonreducing or reducing end within the crystalline cellulose sections; and lastly, β-glucosidase, facilitating the release of glucose from cellobiose [80]. Both *Tetracladium* spp. genomes exhibited the complete repertoire of these enzymes, which were present in multiple copies.

Regarding lignin degradation, the genomes lacked the essential enzymes for lignin degradation. Notably, in the annotation of CAZy families, AA2 is associated with lignin as a substrate (Fig. 2B). This family encompasses secreted heme-containing enzymes that utilise hydrogen peroxide or organic peroxides as electron acceptors, including class II lignin-modifying peroxidases [81]. However, upon a closer inspection of the specific lignin-degrading and modifying enzymes, we found that the genomes lacked the essential enzymes for these processes. The proteins within the AA2 group (*T. maxilliforme*, a single copy; *T. marchalianum*, 3 copies) were identified as cytochrome-C peroxidases (EC 1.11.1.5) based on enzyme numbers, catalysing the degradation of hydrogen peroxide. Moreover, the sole enzyme associated with lignin degradation/modification identified in the genomes is glyoxal oxidase (EC 1.2.3.15), functioning as an oxidoreductase catalysing glyoxal or methylglyoxal reduction or glyoxalase and pyruvate reduction. This is considered auxiliary in lignin degradation as it provides H_2_O_2_ for lignin-degrading oxidases as an electron acceptor in their catalytic cycles [82]. Glyoxal oxidase has also been recognised for its role in catalysing the oxidation of aldehydes and toxins [83]. Originally described in Basidiomycetes in the context of lignin degradation [84], more recent findings in *Vitis pseudoreticulata* indicate its involvement in inducing resistance against *Erysiphe necator* [85]. In the context of these two *Tetracladium* species, glyoxal oxidase may play a role in generating H_2_O_2_, potentially contributing to a defence mechanism against other fungal species. This is supported by the observed co-exclusion relationship between the *Tetracladium* genus and root pathogenic fungi in oilseed rape [14].

### Chitin degradation potential of the genomes

In addition to cellulose degradation, our analysis revealed a significant abundance of CAZymes involved in the degradation of fungal cell walls. Notably, *T. maxilliforme* exhibited a higher proportion of genes related to chitin and glycan degradation, while *T. marchalianum* demonstrated a higher abundance of genes associated with mannan degradation. Fungal cell walls primarily consist of glucan and chitin, with varying additional components among fungal species [86]. Wall-associated enzymes, including chitinases, glucanases, peptidases, and glycosyl-transferases play a crucial role in cell wall synthesis and remodelling [87]. These enzymes are involved in breaking down and synthesising cell wall components [86]; therefore, these enzymes are a natural part of the fungal genome.

However, the biological functions of chitinases are very diverse and include roles in yeast and filament morphogenesis, autolysis, acquisition of chitin for nutritional purposes, and mycoparasitism [88]. Some fungal species, such as *Trichoderma virens*, *Aspergillus nidulans*, *Tolypocladium ophioglossoides*, and *Trichoderma atroviride* are known to have more than one copy of the chitinase genes [89]. However, in addition to CAZymes, proteases, especially aspartic proteases, contribute to fungal cell wall degradation [90]. The *Tetracladium* spp. genomes exhibited high aspartic protease copy numbers, suggesting potential roles of fungal cell wall degrading enzymes beyond cell wall remodelling. The recycling of building blocks from microbial necromass is known to conserve energy, which explains the survival of microbes in deeper soil layers by utilising necromass resources [91]. Our analysis identified all known fungal enzymes involved in the chitin degradation pathway. The exploration of microbial mining explains how microbes in deeper soil layers sustain themselves by relying on the downward flow of necromass, thereby regulating carbon utilisation based on the availability of these resources [92]. This mechanism could potentially be interesting in understanding *Tetracladium*’s ecology in the soil.

Additionally, the genome of *T. maxilliforme* encodes peptidoglycan-degrading enzymes, suggesting a potential for bacterial cell wall degradation and facilitation of bacteria-fungus interactions. However, it is noteworthy that *T. marchalianum* has exhibited antagonistic effects against pathogenic bacteria such as *Agrobacterium tumefaciens*, *Bacillus subtilis*, *Erwinia chrysanthemi*, *Escherichia coli*, and *Xanthomonas phaseoli* [29], while *T. maxilliforme* remains understudied in this context.

### Biosynthetic potential of bioactive secondary metabolites

The production of bioactive secondary metabolites may also mediate antagonism against fungi and potentially other (micro)organisms. Using antiSMASH, we were able to predict the presence of 51 and 22 secondary metabolite BGCs in *T. maxilliforme* and *T. marchalianum*, respectively. The genome of *T. maxilliforme* includes a BGC that has high homology to that of the cyclic depsipeptides aureobasidin A1, which is an antifungal antibiotic [93] and the mycotoxin enniatins, which are known to have various bioactivities including antibiotic, antifungal and insecticidal [94]. It also includes BGCs with high homology to those of the antifungal antibiotic ilicicolin H, which inhibits mitochondrial respiration [95] and ascochlorin, which is a metabolite produced by various fungi and has a wide range of bioactivities such as antiviral, anti-inflammatory and cytotoxic [96]. Similarly, the genome of *T. marchalianum* includes a BGC that shows high homology to that of xenortide A, which is known to have antimicrobial activity against *Plasmodium falciparum* and *Trypanosoma brucei* [97]. Experimental validation would be needed to confirm the production of such metabolites in *T. maxilliforme* and *T. marchalianum*. Interestingly, aside from those mentioned above, the majority of the BGCs found in both fungal genomes showed either limited similarity to known BGCs or no homology to any known BGCs at all, which suggests potential for novel secondary metabolite production in these strains.

### Further evidence for versatile ecologies

The clustering of *Tetracladium* spp. genomes with those of endophytes or genomes associated with multiple ecological roles in the context of peptidases and lipases further supports their ecological versatility. However, this clustering did not distinctly delineate between the multiple ecological and saprotrophic lifestyles observed in *T. maxilliforme* and *T. marchalianum*, as highlighted by their CAZyme profiles. The comparative examination of lipase profiles revealed distinct clustering patterns, suggesting potential differences in lipid metabolism and ecological functions between *T. maxilliforme* and *T. marchalianum*.

The UMAP plot depicting transporters illustrates commonalities among *Tetracladium* spp. genomes, which formed a distinct cluster in conjunction with other endophytic or ecologically diverse genomes. Notably, most endophytes, except *Xylona heveae*, shared this distinct grouping. *Xylona heveae*, isolated from *Hevea brasiliensis* [98], lacks comprehensive information regarding its ecological versatility. While it has been suggested that the two *Neurospora* spp., which were not part of this lipase cluster, are plant-associated [99], there is little supporting evidence and they are most likely free-living saprotrophs [100]. Finally, *Wickerhamomyces anomalus*, a saprotrophic yeast and opportunistic human pathogen [101] remains insufficiently studied, with its lifestyle not well characterized. Hence, comparative analysis of *Tetracladium* transporter profiles proved to be insufficient as an indicator of lifestyle.

As obligate symbionts, Glomeromycotan arbuscular mycorrhizal fungi are thought to depend on critical materials provided by their host plants with studies highlighting the significance of fatty acids and thiamine auxotrophy [102–104]. Fungi possess two sets of FAS genes: Type I FAS and Type II FAS [105]. Type I FAS comprises cytosolic genes responsible for the production of long-chain fatty acids, while type I FAS is typically encoded by a single octa-functional gene in animals and most Basidiomycota. Ascomycota harbour two tetra-functional type I FAS genes, FAS1 and FAS2 [106]. In contrast, type II FAS genes resemble bacteria-like genes, which are responsible for synthesising the mitochondrial respiratory cofactor lipoic acid [107]. Some studies suggest that arbuscular mycorrhizal fungi are missing Type I FAS genes from their genomes while the majority of Type II FAS genes are present [70, 72]. Our analysis revealed that both *Tetracladium* genomes contain FAS1 and FAS2, indicating the presence of a complete cytosolic FAS pathway. This finding contrasts with the metabolic dependency observed in Glomeromycotan fungi suggesting that *Tetracladium* may possess an independent capability for fatty acid biosynthesis.

Glomeromycotan mycorrhizal fungi also lack genes in the thiamine biosynthesis pathway, serving as a potential indicator for symbiotic dependency [69]. We found that some genes of the pathway were missing from the *Tetracladium* genomes. Our investigation identified gaps in fatty acid synthesis and thiamine biosynthesis genes in *Tetracladium* species.

## Conclusions

Our findings enhance our understanding of the ecological versatility and functional capabilities of *Tetracladium* spp., laying the groundwork for future studies on their ecological interactions and biotechnological applications. However, while comparative genomics offers valuable insights into the functional annotations and genomic diversity among organisms, it comes with certain limitations. Many genomes, especially those of non-model organisms or microorganisms, may have incomplete or fragmented sequences. This can limit the accuracy and comprehensiveness of comparative analyses. Computational methods used for gene prediction may not always accurately identify all genes within a genome, leading to potential underestimation or overestimation of gene content. Assigning functions to genes based solely on sequence homology can be challenging, particularly for genes with no known homologs or those involved in novel pathways.

## Supplementary Information

Supplementary Material 1.

Supplementary Material 2.

Supplementary Material 3.

Supplementary Material 4.

Supplementary Material 5.

Supplementary Material 6.

Supplementary Material 7.

Supplementary Material 8.

Supplementary Material 9. Supplementary Table 1. – Metadata information for the 41 Ascomycotan genomes. Supplementary Table 2. – Genes associated with fatty acid (FAS) and thiamine synthesis and their presence in the *Tetracladium maxilliforme* and *Tetracladium marchalianum* genomes. Supplementary Table 3. – Details of biosynthetic gene clusters (BGCs) present in each of the two genomes reported in this study, including BGC location, class, span, size and most similar known BGC. Supplementary Table 4. – Comparison of BUSCO databases for completeness assessment. Supplementary Figure 1. – Hierarchical clustering analysis on the*Tetracladium maxilliforme* (Tetracladium max), *Tetracladium marchalianum* (Tetracladium march) and the Ascomycotan genomes. The clustering was based on the abundance and composition of carbohydrate-active enzyme (CAZyme) classes. The CAZyme categories are glycoside hydrolases (GH), carbohydrate esterases (CE), carbohydrate-binding modules (CBM), polysaccharide lyases (PL), auxiliary activities enzymes (AA), and glycosyl transferases (GT). Shading darkness shows protein copy numbers. Coloured boxes next to the short genome names show lifestyle. Supplementary Figure 2. – Hierarchical clustering analysis on the*Tetracladium maxilliforme* (Tetracladium max), *Tetracladium marchalianum* (Tetracladium march) and the Ascomycotan genomes. The clustering was based on the abundance and composition of peptidases. Shading darkness shows protein copy numbers. Coloured boxes next to the short genome names show lifestyle. Supplementary Figure 3. – Hierarchical clustering analysis on the*Tetracladium maxilliforme* (Tetracladium max), *Tetracladium marchalianum* (Tetracladium march) and the Ascomycotan genomes. The clustering was based on the abundance and composition of lipases. Shading darkness shows protein copy numbers. Coloured boxes next to the short genome names show lifestyle. Supplementary Figure 4. – A – Uniform Manifold Approximation and Projection (UMAP) ordination plots of the transporter profiles of the genomes. The colour of the circles shows lifestyles. B – UMAP ordination plots of the small secreted protein profiles of the genomes. The colour of the circles denotes lifestyle

## Data Availability

*Tetracladium maxilliforme* and *Tetracladium marchalianum* assembled genomes can be found under BioProject PRJNA1063930. Functional annotations of the genomes are available in FigShare under: *T. maxilliforme*: 10.6084/m9.figshare.28239119.v2 and *T. marchalianum*: 10.6084/m9.figshare.28239131.v1.
