# Supplementary figures and images for: Genomic insights into the ecological versatility of Tetracladium spp
Source: BMC Genomics. 2025 Nov 5;26:998. doi: 10.1186/s12864-025-12146-z (PMC12590662; doi:10.1186/s12864-025-12146-z)

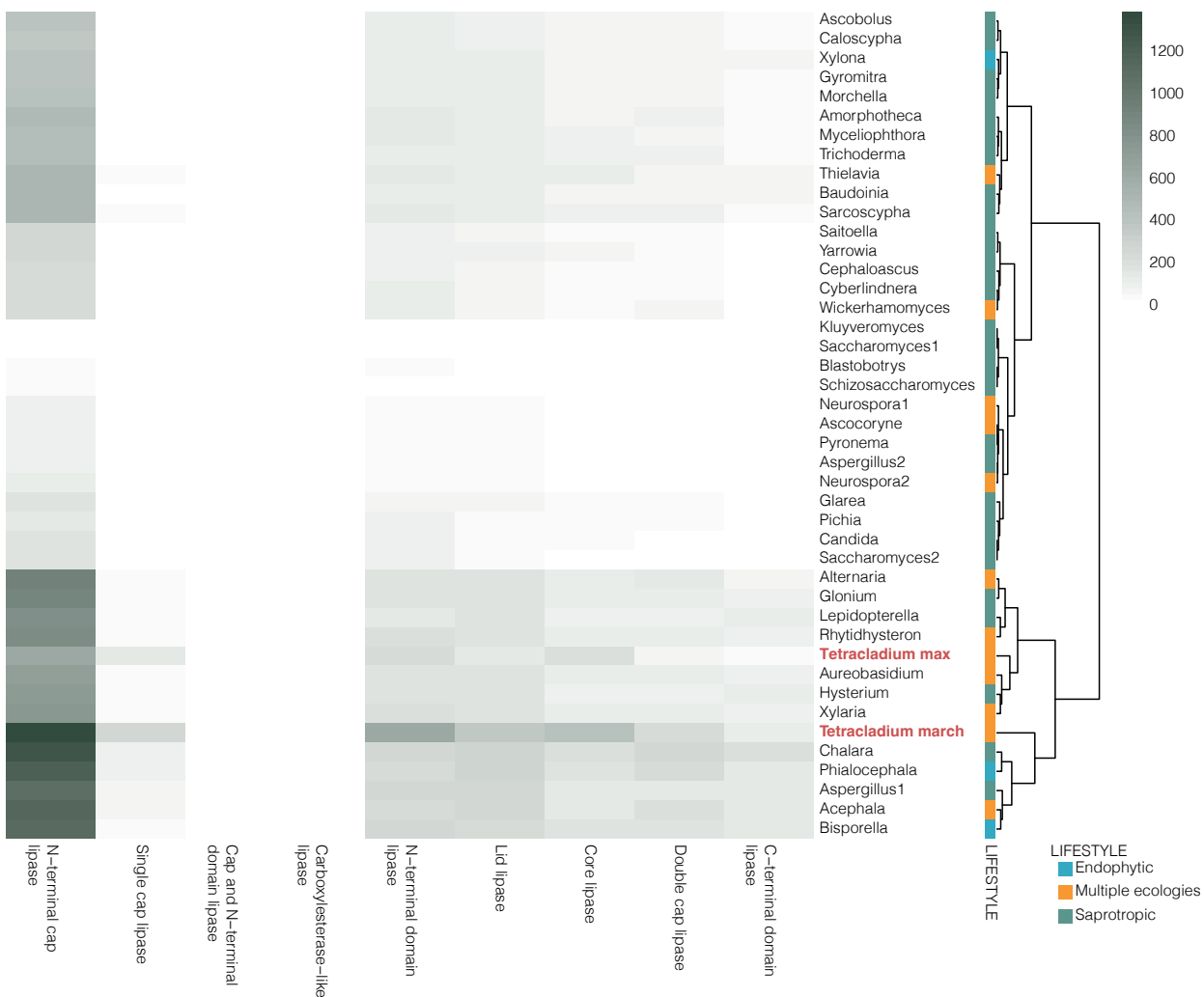

Supplement: Supplementary file 3 — Supplementary Material 3. [file 12864_2025_12146_MOESM3_ESM.pdf]

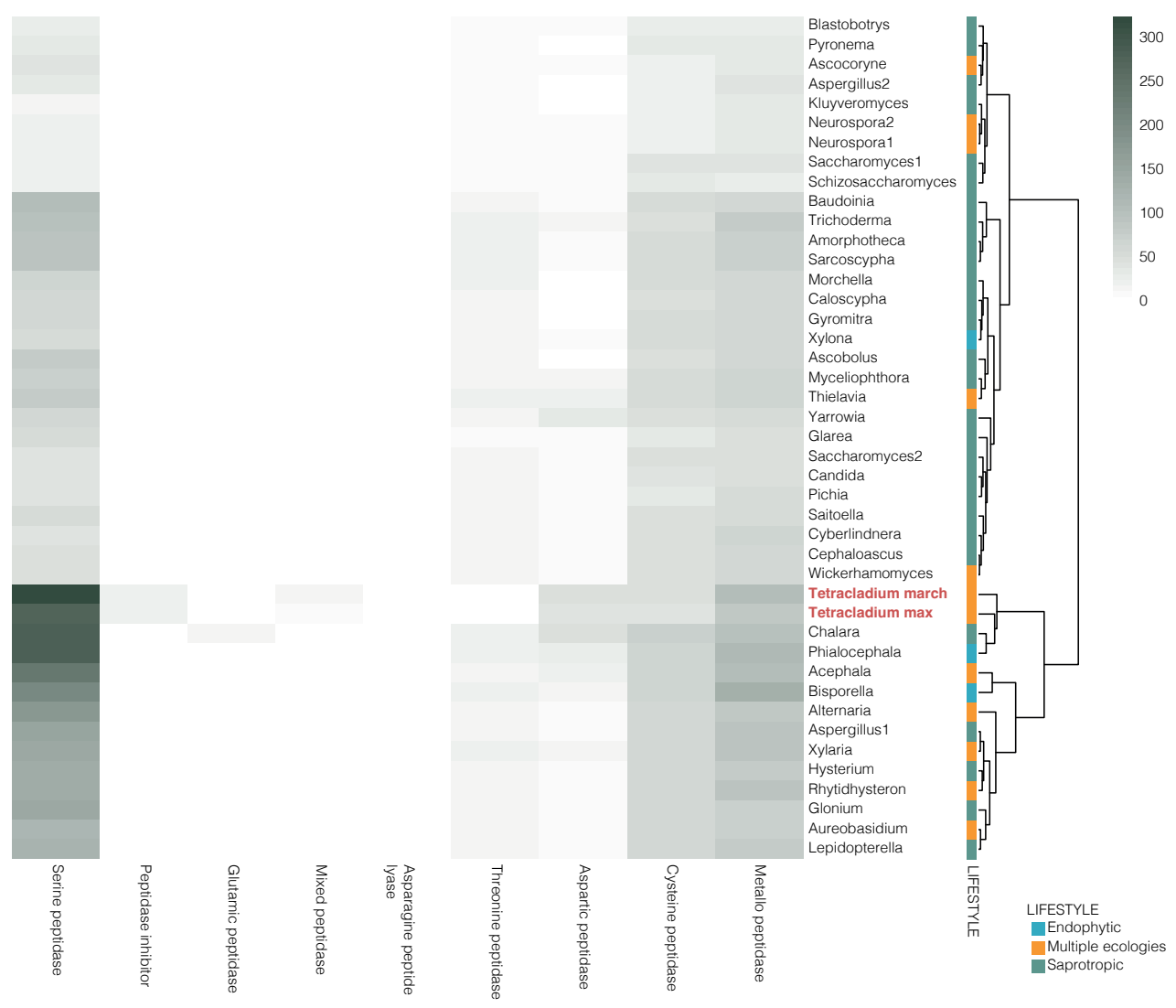

Supplement: Supplementary file 4 — Supplementary Material 4. [file 12864_2025_12146_MOESM4_ESM.pdf]

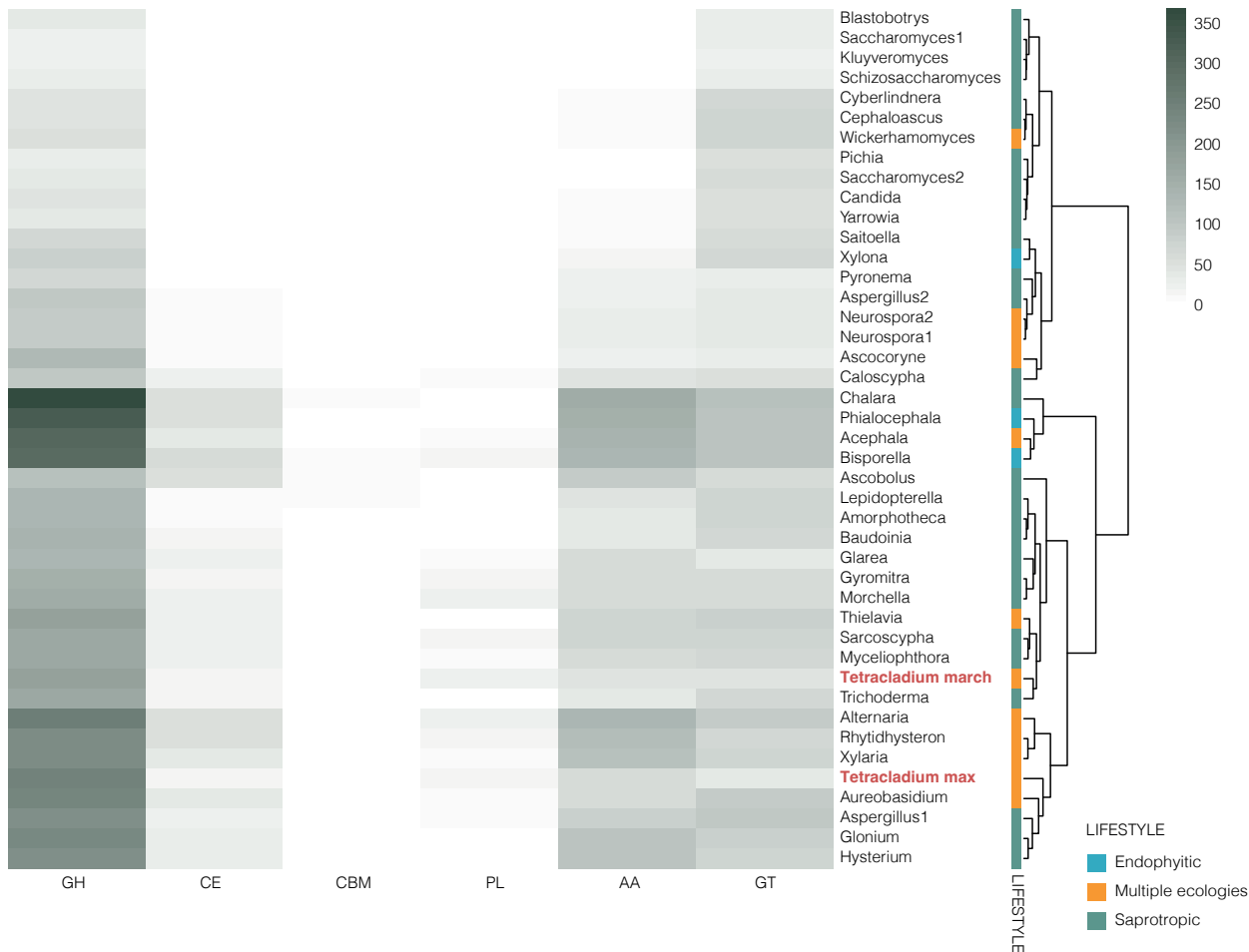

Supplement: Supplementary file 5 — Supplementary Material 5. [file 12864_2025_12146_MOESM5_ESM.pdf]

A

## TRANSPORTERS

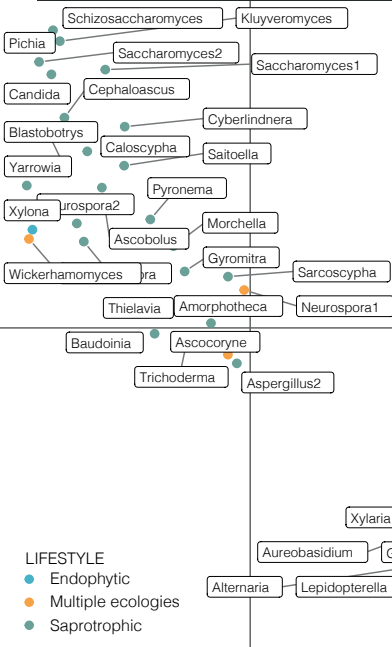

B

## SMALL SECRETED PROTEINS

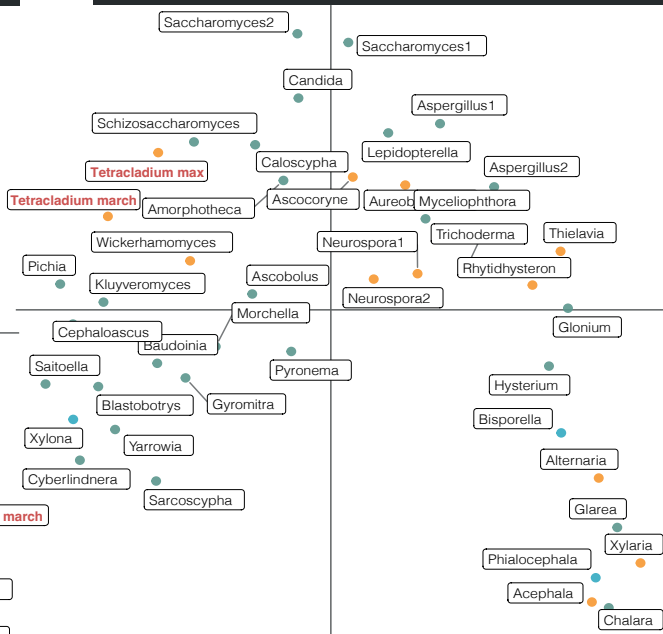

Supplement: Supplementary file 6 — Supplementary Material 6. [file 12864_2025_12146_MOESM6_ESM.pdf]
