# Supplementary material for: Genomic insights into the ecological versatility of Tetracladium spp
Source: BMC Genomics. 2025 Nov 5;26:998. doi: 10.1186/s12864-025-12146-z (PMC12590662; doi:10.1186/s12864-025-12146-z)
Supplement: Supplementary file 9 — Supplementary Material 9. Supplementary Table 1. – Metadata information for the 41 Ascomycotan genomes. Supplementary Table 2. – Genes associated with fatty acid (FAS) and thiamine synthesis and their presence in the Tetracladium maxilliforme and Tetracladium marchalianum genomes. Supplementary Table 3. – Details of biosynthetic gene clusters (BGCs) present in each of the two genomes reported in this study, including BGC location, class, span, size and most similar known BGC. Supplementary Table 4. – Comparison of BUSCO databases for completeness assessment. Supplementary Figure 1. – Hierarchical clustering analysis on theTetracladium maxilliforme (Tetracladium max), Tetracladium marchalianum (Tetracladium march) and the Ascomycotan genomes. The clustering was based on the abundance and composition of carbohydrate-active enzyme (CAZyme) classes. The CAZyme categories are glycoside hydrolases (GH), carbohydrate esterases (CE), carbohydrate-binding modules (CBM), polysaccharide lyases (PL), auxiliary activities enzymes (AA), and glycosyl transferases (GT). Shading darkness shows protein copy numbers. Coloured boxes next to the short genome names show lifestyle. Supplementary Figure 2. – Hierarchical clustering analysis on theTetracladium maxilliforme (Tetracladium max), Tetracladium marchalianum (Tetracladium march) and the Ascomycotan genomes. The clustering was based on the abundance and composition of peptidases. Shading darkness shows protein copy numbers. Coloured boxes next to the short genome names show lifestyle. Supplementary Figure 3. – Hierarchical clustering analysis on theTetracladium maxilliforme (Tetracladium max), Tetracladium marchalianum (Tetracladium march) and the Ascomycotan genomes. The clustering was based on the abundance and composition of lipases. Shading darkness shows protein copy numbers. Coloured boxes next to the short genome names show lifestyle. Supplementary Figure 4. – A – Uniform Manifold Approximation and Projection (UMAP) ordinati [file 12864_2025_12146_MOESM9_ESM.docx]

**Supplementary Table 1.** – Metadata information for the 41 Ascomycotan genomes.

**Supplementary Figure 1.** – Hierarchical clustering analysis on the *Tetracladium maxilliforme* (Tetracladium max), *Tetracladium marchalianum* (Tetracladium march) and the Ascomycotan genomes. The clustering was based on the abundance and composition of carbohydrate-active enzyme (CAZyme) classes. The CAZyme categories are glycoside hydrolases (GH), carbohydrate esterases (CE), carbohydrate-binding modules (CBM), polysaccharide lyases (PL), auxiliary activities enzymes (AA), and glycosyl transferases (GT). Shading darkness shows protein copy numbers. Coloured boxes next to the short genome names show lifestyle.

**Supplementary Figure 2.** – Hierarchical clustering analysis on the *Tetracladium maxilliforme* (Tetracladium max), *Tetracladium marchalianum* (Tetracladium march) and the Ascomycotan genomes. The clustering was based on the abundance and composition of peptidases. Shading darkness shows protein copy numbers. Coloured boxes next to the short genome names show lifestyle.

**Supplementary Figure 3.** – Hierarchical clustering analysis on the *Tetracladium maxilliforme* (Tetracladium max), *Tetracladium marchalianum* (Tetracladium march) and the Ascomycotan genomes. The clustering was based on the abundance and composition of lipases. Shading darkness shows protein copy numbers. Coloured boxes next to the short genome names show lifestyle.

**Supplementary Figure 4.** – A – Uniform Manifold Approximation and Projection (UMAP) ordination plots of the transporter profiles of the genomes. The colour of the circles shows lifestyles. B – UMAP ordination plots of the small secreted protein profiles of the genomes. The colour of the circles denotes lifestyle.

**Supplementary Table 2.** – Genes associated with fatty acid (FAS) and thiamine synthesis and their presence in the *Tetracladium maxilliforme* and *Tetracladium marchalianum* genomes.

**Supplementary Table 3.** – Details of biosynthetic gene clusters (BGCs) present in each of the two genomes reported in this study, including BGC location, class, span, size and most similar known BGC.

**Supplementary Table 4.** – Comparison of BUSCO databases for completeness assessment.
